# Author’s response: Critical need for robust surveillance in response to DENV-2 and SARS-CoV-2 cross-reactivity

**DOI:** 10.2807/1560-7917.ES.2024.29.19.2400270

**Published:** 2024-05-09

**Authors:** Raphaëlle Klitting, Xavier de Lamballerie, on behalf of all co-authors of the original article

**Affiliations:** 1 National Reference Center for Arboviruses, Inserm-IRBA, Marseille, France; 2 Unité des Virus Émergents (UVE: Aix-Marseille Univ, Università di Corsica, IRD 190, Inserm 1207, IRBA), Marseille, France; 3 https://www.eurosurveillance.org/content/10.2807/1560-7917.ES.2024.29.13.2400123

**Keywords:** dengue fever, surveillance, pathogenesis

**To the editor**: We would like to thank the authors of the letter “Critical need for robust surveillance in response to DENV-2 and SARS-CoV-2 cross-reactivity” [1], for their interest in our work [2] and for bringing up the question of the impact of the co-circulation of dengue virus 2 (DENV-2) and severe acute respiratory syndrome coronavirus 2 (SARS-CoV-2) in terms of public health. The joint circulation of these two viruses raises a number of important issues for human health, including the pressure on healthcare systems, the risk of co-infections and of potential immunopathological interactions.

In their letter, the authors refer to a recent preprint titled “SARS-CoV-2 antibodies cross-react and enhance dengue infection” that investigates the enhancement of DENV2 infection in vitro with cross-reactive SARS-CoV-2 antibodies acquired from natural infection in humans, through experimental immunisation in animals, or available commercially [3]. This is a very preliminary step before demonstrating the clinical implication of an antibody dependent enhancement (ADE) phenomenon of DENV2 infection by SARS-CoV-2 antibodies. Of note, the study assesses a set of convalescent sera collected in India during the pre-/post-Delta and Omicron waves of SARS-CoV-2. Since India is endemic for DENV and as the authors do not report a strategy for excluding sera from patients with a history of dengue infection, it is difficult to establish whether the results observed are actually due to cross-reactive antibodies against SARS-CoV-2 or antibodies against dengue from prior infection. Further, the absence of association between neutralising antibody titre and ADE efficiency reported by the authors could also be explained by the presence of anti-DENV antibodies from a past infection.

More importantly, and beyond the results of the study itself, the demonstration of an ADE phenomenon in vitro is not sufficient to conclude that antibody-dependent disease enhancement occurs in vivo, and even less so in real-life conditions [4]. While previous studies have found evidence of ADE in vitro for several viruses including influenza, HIV, and Ebola virus, no involvement in pathogenesis could ever be demonstrated in vivo or in clinical studies for these infections [5]. Specifically for SARS-CoV-2, it has been shown that natural infection commonly elicits both neutralising antibodies and antibodies demonstrating in vitro enhancement of virus infection [6,7], but even the latter afforded protection in animal models and to date, to the best of our knowledge, ADE has not been shown to be part of the pathological mechanism of severe COVID-19. This does not imply that the opposite mechanism (involvement of anti-SARS-CoV-2 antibodies in facilitating infection by the dengue virus) is not possible, but it puts into perspective the relationship between the detection of facilitating antibodies in vitro and the existence of an associated severe clinical form.

In conclusion, the question of a potential immunopathological interaction between dengue and SARS-CoV-2 infection requires the production of robust preclinical, epidemiological and clinical information to evaluate the importance of SARS-CoV-2 antibodies that cross-react with DENV2 in vitro and, in fine, inform diagnostics, patient management, and control strategies at the population level.
